# Interrelationship Between Age, Gender, and Weight Status on Motor Coordination in Italian Children and Early Adolescents Aged 6–13 Years Old

**DOI:** 10.3389/fped.2021.738294

**Published:** 2021-09-10

**Authors:** Giuseppe Battaglia, Valerio Giustino, Garden Tabacchi, Massimo Lanza, Federico Schena, Valentina Biino, Matteo Giuriato, Maria Chiara Gallotta, Laura Guidetti, Carlo Baldari, Antonino Gennaro, Antonio Palma, Marianna Bellafiore

**Affiliations:** ^1^Sport and Exercise Sciences Research Unit, Department of Psychology, Educational Science and Human Movement, University of Palermo, Palermo, Italy; ^2^Department of Neuroscience, Biomedicine and Movement, University of Verona, Veneto, Italy; ^3^Department of Physiology and Pharmacology “Vittorio Erspamer”, Sapienza University of Rome, Rome, Italy; ^4^Department Unicusano, University Niccolò Cusano, Rome, Italy; ^5^Department of Theoretical and Applied Sciences, eCampus University, Rome, Italy; ^6^Department of Biomedical, Dental Sciences and Morphofunctional Imaging, AOU “G. Martino”, University of Messina, Messina, Italy

**Keywords:** motor coordination, motor performance, motor quotient, weight status, body weight, overweight, obesity, children

## Abstract

Although numerous evidences reported a negative correlation between motor coordination (MC) and overweight/obesity in children and adolescents, the interrelationship between age, gender, and weight status is still debatable. Hence, the aim of this cross-sectional study was to examine the association between MC and weight status according to age and gender across childhood and early adolescence in a large sample of Italian elementary and middle school students. A number of 1961 Italian school students (1,026 boys, 935 girls) was stratified in three consecutive age groups (6–7, 8–10, and 11–13 years) and four weight status categories (underweight, normal weight, overweight, and obese) according to Cole's body mass index (BMI) cut-off points for children. MC performance was assessed measuring motor quotient (MQ) with the Körperkoordinationstest für Kinder (KTK). Results showed significantly lower MQ levels in children in overweight (OW) and with obesity (OB) in both sexes for all age groups than peers in normal weight (NW), except in 6–7-year-old boys. Girls in OW and with OB had similar MQ levels across all age groups, while younger boys in OW and with OB showed higher MQ levels than older ones (*p* < 0.05). The 6–7-year-old boys showed better MQ levels than girls peers in NW, OW, and with OB, while 8–10-year-old boys in underweight (UW), NW, and OW; and 11–13-year-old boys only in NW (*p* < 0.05). No interaction effect was found between age, gender, and weight status on MQ levels. These outcomes showed the negative impact of higher weight status on MC performance according to age and gender, pointing out the importance of planning targeted motor programs that consider these variables to improve MC performance.

## Introduction

Motor coordination (MC), or motor competence, is a term that describes the ability to perform both fine and gross motor skills (1, 2). It is widely recognized that an adequate level of MC in children is fundamental to develop specialized sequences of movement for daily life tasks and for organized and non-organized physical activities. For this reason, it is crucial that a high level of MC is developed during the preschool and primary school period, to be perfected later (1, 3–6).

Furthermore, the scientific literature has highlighted the role of MC development on the consequent childhood obesity prevention and, similarly, several studies have highlighted the influence of body weight status on MC performance (7–11). The development of MC in children may be an important contributing factor to negative or positive weight trajectories (9, 12, 13). The importance of an adequate development in motor skills is also given by the fact that previous studies reported that childhood obesity involves not only locomotion and object control skills but also the ability in executing basic daily life activities (14).

Moreover, there is a body of studies in this field that have emphasized the relationship between MC and the practice of physical activity (PA) (15–17). As a matter of fact, D'Hondt et al. (9) showed that the associations between PA, MC, and body weight generally increase in childhood (9, 13).

In this perspective, the relationship between MC and weight status has been extensively studied in children and adolescents in overweight (OW) and with obesity (OB) showing impaired development of motor skills (18–21). To the best of our knowledge, an inverse relationship exists between body mass index (BMI) and MC in childhood and adolescence with similar associations between girls and boys over time (9, 12, 13, 21).

Although evidences suggest a negative correlation between MC performance and excess body mass (22–24), this relationship still remains debatable. In particular, there is a body of articles suggesting that children and adolescents in OW and with OB show lower level of MC than their peers in healthy-weight, regardless of age (21, 25–29).

Some research groups have investigated the association between children's body weight status and gross motor performances based on gender, reporting different results (29, 30). For instance, Cawley et al. (31) reported reduced MC only in boys with OB, while no differences were found among girls with OB girls and peers in normal weight (NW) (31).

The prevalence of children in OW and with OB is increasing worldwide, representing one of the most serious public health conditions nowadays and, as stated above, this weight status is related to sedentary behaviors and consequent lower MC proficiency (11, 32, 33). In detail, in Italy the prevalence of children in OW and with OB is 22.5 and 9.3%, respectively (34).

Therefore, the purpose of this study was to analyse the association between MC, assessed by the Körperkoordinationstest für Kinder (KTK), and weight status in 6– to 13-year-old Italian boys and girls. Moreover, we investigated the association between performances of KTK subtests (i.e., walking backward, jumping sideways, hopping for height, and moving sideways), and weight status according to age and gender.

Although some researches have investigated MC performance in different Italian regions during childhood and adolescence (26, 35), this study is the first to consider a large sample from all over the nation and taking into account the influence of weight status, age, and gender. The novelty of the study is not only the large sample recruited, but also the investigation of all subtest performances according to weight status, age, and gender.

We hypothesized that weight status similarly affects the levels of MC in girls and boys. In particular, children and adolescents with higher BMI (i.e., in OW and with OB) should show lower levels of MC than their peers in NW. Furthermore, we expected to find higher levels of motor competence in boys than girls, and in adolescents than children. As for the subtest performances, we expected that body weight could affect differently depending on the specificity of each subtest, that is, we hypothesized to find scores gradually decreasing with increasing body weight in motor skills that required greater effort to counteract the force of gravity (i.e., jumping sideways and hopping for height), regardless of age and gender.

## Materials and Methods

### Participants

A number of 1,961 participants (boys: *n* = 1,026; girls: *n* = 935), between 6 and 13 years of age (Table 1) were recruited from elementary and middle schools in northern, central, and southern Italy, chosen as widely representative schools of the three Italian geographical areas. The measurements of this cross-sectional study were conducted in the participating schools from January 2019 to February 2020 during the regular school hours and in the respective school gyms.

**Table 1:** Descriptive statistics for weight status categories and gender classes stratified by age groups.

			**Height (m)**		**Weight (kg)**		**BMI (kg/m^2^)**		**UW**		**NW**		**OW**		**OB**	
**Age (years)**	***n***	**%**	**Mean**	**SD**	**Mean**	**SD**	**Mean**	**SD**	***n***	**%**	***n***	**%**	***n***	**%**	***n***	**%**
**Boys**																
6–7	242	24	1.25	0.70	26.0	5.67	16.6	2.50	26	31	157	25	38	17	21	23
8–10	522	50	1.39	0.08	36.3	9.33	18.6	3.60	40	47	293	47	129	58	60	65
11–13	262	26	1.55	0.11	46.9	11.33	19.3	3.29	19	22	175	28	57	25	11	12
Tot	**1026**	**100**	**1.40**	**0.14**	**36.6**	**11.76**	**18.3**	**3.44**	**85**	**100**	**625**	**100**	**224**	**100**	**92**	**100**
**Girls**																
6–7	261	28	1.23	0.07	25.6	5.23	16.8	2.60	27	33	146	26	62	31	26	29
8–10	489	52	1.38	0.09	35.3	9.87	18.5	3.73	43	53	276	49	109	55	61	67
11–13	185	20	1.54	0.08	46.1	9.17	19.2	2.84	11	14	143	25	27	14	4	4
Tot	**935**	**100**	**1.37**	**0.13**	**34.8**	**11.12**	**18.2**	**3.39**	**81**	**100**	**565**	**100**	**198**	**100**	**91**	**100**
**TOT**																
	**1961**	**100**	**1.38**	**0.14**	**35.7**	**11.50**	**18.2**	**3.42**	**166**	**8**	**1190**	**61**	**422**	**22**	**183**	**9**

*n, number; %, percentage frequency; SD, standard deviation; UW, underweight; NW, normal weight; OW, overweight; OB, obesity. Total values in bold*.

This multicentre study has been led by department of Neuroscience, Biomedicine and Movement (University of Verona) and approved by the Ethical Board of Verona University (No. 2019-UNVRCLE-0298910) and Palermo University (No. 8/2019). The study complies with the criteria for the use of people in research defined in the Declaration of Helsinki. Moreover, school principals/administrators provided further authorizations for the study. After researchers explained the purpose of the investigation and the research methodology, all parents provided written informed consent prior to participating in the study.

### Anthropometric Measurements

As for the anthropometric measurements, participants' body weight (kg) and height (m) were measured using an electronic scale and a standard audiometer to the nearest 0.5 kg and 0.1 cm, respectively. Body mass index (BMI) was computed as body weight divided by height squared (kg/m^2^). All measures were collected by examiners who were trained in the measurement methods of height and weight.

According to the Cole's BMI percentiles for boys and girls aged 2–18 years (36), the following four categories of weight status were obtained: underweight (UW) below the 5th percentile, normal weight (NW) between 5th and 85th percentile, overweight (OW) between 85th and 95th percentile, and obesity (OB) over the 95th percentile.

### Motor Coordination Assessment

Motor coordination (MC) was measured using the Körperkoordinationstest für Kinder (i.e., a body coordination test for children, referred to as KTK) by examiners who were trained in the administration of the KTK (37, 38). The KTK is a standardized, norm-referenced measure for age and gender that allows to assess MC, expressed as motor quotient (MQ), in children aged 5–14 years. The KTK was administered and scored according to the manual guidelines.

The test protocol included four subtests: (1) walking backward (WB) on a balance beam of 3 m in length of decreasing widths (6, 4.5, and 3 cm); (2) jumping sideways (JS) on two feet from side to side over a small beam (60 × 4 × 2cm) as fast as possible for 15 s; (3) hopping for height (HH) on one foot over a foam obstacle of increasing height (consecutive increments of 5 cm); (4) moving sideways (MS) on the floor in 20 s by stepping from one plate (25 × 25 × 2 cm, supported on four legs 3.7 cm high) to the next, moving onto the first plate, step ping on it, and so on.

The total MQ, standardized for age and gender, was calculated starting from the raw scores of each subtest, according to normative data tables provided by the manual. The total MQ was calculated by adding the four subtest scores. As indicated by Kiphard and Schilling (2007) KTK showed acceptable construct validity (38). In the test-retest for the raw score on the total test battery the reliability coefficient was 0.97, while corresponding coefficients for each subtest ranged from 0.80 to 0.96 (38).

### Statistical Analysis

For statistical analyses, participants were divided according to the four weight status categories aforementioned, three consecutive age groups (6–7, 8–10, and 11–13 years), and gender classes.

The Shapiro-Wilk test for normality was initially used to evaluate the distribution of data. Means and Standard Deviations (SD) of weight, height, and BMI were calculated. Number and percentage frequencies were displayed to describe the weight status by age and gender. The Chi-Square test was carried out to study any significance between frequencies of weight status categories according to age groups. The scores of the KTK performance were calculated and showed as means and SDs by age groups, gender classes, and weight status categories.

The one-way Analysis of Variance (ANOVA) with Bonferroni's correction was initially performed separately between the MQ/subtest scores and weight status, age, and gender to explore the influence of each of these three variables. In order to examine any significant difference across weight status categories according to age groups and gender classes, across age groups according to weight status categories and gender classes, and across gender classes according to weight status categories and age groups, the one-way ANOVA analyses were subsequently performed. The results of the one-way ANOVA were displayed through the *F*-values and level of significance, which was set at *p* < 0.05.

The three-way ANOVA was run to examine if there was an interaction effect between the independent variables weight status, age, and gender on MQ and subtests scores. The interaction results were showed as partial sum of squares (SS) that helps express the total variation that can be attributed to the single factors and to the interaction of factors, and degrees of freedom (df), *F*-values, and level of significance were also provided. The adjusted predictions of weight status, age, and gender on MQ were plotted in a graph.

The software STATA/MP 12.1 (StataCorp LP, College Station, TX, USA) was used to perform the statistical analyses.

## Results

### Characteristics of the Participants

Descriptive statistics of the participants are presented in Table 1, which shows data on the frequency of boys and girls in UW, NW, OW, and with OB categories by age groups (6–7, 8–10, 11–13 years old). The prevalence of UW, NW, OW, and with OB was 8, 61, 22, and 9%, respectively. In particular, the prevalence of participants in OW was significantly higher (*p* < 0.05) in 6–7-year-old girls than boys of the same age range (31 vs. 17%), while it was significantly lower (*p* < 0.05) in 11–13-year-old boys than girls' peers (14 vs. 25%). Higher prevalence of participants with OB was found out in boys aged 11–13-year-old than girls in the same age range (12 vs. 4%), but this difference was not significant.

### Analyses of Motor Coordination Levels by Weight Status, Age, and Gender

The one-way ANOVA between MQ and weight status, age, and gender revealed that these three variables were strongly associated with MQ. In detail, MQ level was progressively reduced (*p* < 0.001) in: (1) weight status categories from UW/NW (in these two categories performances were not different) to OW and OB (*F* = 37.66); (2) older than younger ones (*F* = 25.25); (3) girls than boys (*F* = 67.22) (Table 2). The same trend was evidenced for the MS and HH subtests, with MS showing a very high significance (*F* = 128.41) across age groups, and HH across gender categories (*F* = 53.84). A significant (*p* < 0.001) inverse trend was revealed for the WB subtest, with performance significantly increasing in older participants (*F* = 12.64) and in girls (*F* = 17.47), while scores were decreasing in OW and OB categories (*F* = 40.38). No differences were found for the JS subtest across age groups, while decreasing performance was found out for girls (*F* = 268.38) and higher weight status categories (*F* = 15.82) (Table 2).

**Table 2:** Total MQ and subtest scores of the KTK by age groups, gender classes, and weight status categories, with F-values and significance levels across the single categories.

	**MQ**	**WB**	**JS**	**MS**	**HH**
6–7	90.2	88.4	99.5	87.4	94.6
8–10	86.6	86.7	98.7	82.5	91.1
11–13	83.3	91.2	100.2	70.9	86.5
F (*d.f*. = 2)	25.25^***^	12.64^***^	1.18	128.41^***^	21.19^***^
Boys	89.3	86.9	105.0	81.6	93.8
Girls	83.7	89.9	93.0	79.7	87.4
F (*d.f*. = 1)	67.22^***^	17.47^***^	268.38^***^	5.55^*^	53.84^***^
UW	88.3	89.4	98.5	83.6	92.7
NW	88.7	91.3	101.2	81.2	91.8
OW	82.5	84.3	95.9	78.5	87.4
OB	77.4	78.6	93.2	77.2	81.6
F (*d.f*. = 3)	37.66^***^	40.38^***^	15.82^***^	5.71^***^	15.98^***^

*MQ, motor quotient; WB, walking backward; JS, jumping sideways; MS, moving sideways; HH, hopping for height; UW, underweight; NW, normal weight; OW, overweight; OB, obesity; d.f., degrees of freedom;*

* *p <0.05;*

^**^p < 0.01;

*** *p <0.001. F-values were estimated through the one-way ANOVA*.

### Analyses of Motor Coordination Levels Across Weight Status Categories by Age and Gender

Significant differences were found out across weight status categories (decreasing performances from participants in OW to participants with OB), both in boys and girls and in all the age groups except for 6–7-year-old boys (Table 3). The strongest significant differences (*p* < 0.001) were evinced across the age groups 8–10 and 11–13 in boys (*F* = 13.45 and *F* = 10.61, respectively), and across the age group 8–10 in girls (*F* = 14.17); while the weakest changes in performance were revealed in age groups 6–7 and 11–13 in girls (*F* = 4.86, *p* < 0.01, and *F* = 2.81, *p* < 0.05, respectively). In most cases, performances in participants in UW were very close to those in NW, while in few cases they were different but non-statistically significant: in 11–13-year-old boys they were higher (mean 92.9 vs. 87.5, *p* > 0.05), and in 8–10 and 11–13-year-old girls they were lower (mean 82.5 vs. 87.0, and 73.8 vs. 81.3, *p* > 0.05, respectively). Subgroups analyses for subtests' scores (Table 3) revealed that the trend was similar to the MQ results, except for the WB and JS subtests that were not significantly different across girls in age groups 11–13 and 6–7, respectively. With regard to the MS subtest, no difference was evidenced across age groups 6–7 and 11–13 years in girls. Contrarily, a significant decreasing performance in the HH subtest was evidenced in boys within the age group 6–7 (*F* = 4.56, *p* < 0.01); while no difference was revealed in boys and in girls in the age group 11–13.

**Table 3:** Total MQ and subtest scores of the KTK by age groups, gender classes, and weight status categories, with *F*-values and significance levels across the weight status categories.

				**Boys**										**Girls**										
				**MQ**		**WB**		**JS**		**MS**		**HH**			**MQ**		**WB**		**JS**		**MS**		**HH**	
**Age groups**	**Weight status categories**	**N TOT**	**N Boys**	**Mean**	**SD**	**Mean**	**SD**	**Mean**	**SD**	**Mean**	**SD**	**Mean**	**SD**	**N Girls**	**Mean**	**SD**	**Mean**	**SD**	**Mean**	**SD**	**Mean**	**SD**	**Mean**	**SD**
6–7	UW	53	26	95.4	15.11	87.0	15.60	102.9	15.26	91.6	17.49	104.5	14.28	27	87.6	14.76	93.7	14.75	91.6	15.34	88.0	14.54	88.5	19.84
	NW	303	157	95.8	14.39	88.9	16.38	104.8	16.00	91.1	15.35	102.5	14.12	146	88.3	16.34	90.5	15.43	96.3	18.83	87.9	15.66	89.3	18.37
	OW	100	38	91.8	13.59	83.1	17.35	106.8	17.63	86.5	13.74	98.6	12.74	62	81.3	13.33	87.0	13.79	90.4	15.42	83.6	14.96	81.5	16.29
	OB	47	21	88.0	14.19	84.0	16.14	104.1	14.59	83.4	12.90	91.7	15.83	26	78.9	14.41	81.9	13.39	89.4	16.44	84.5	15.75	79.5	15.75
	F (*d.f*. = 3)			2.33		1.63		0.34		2.37		4.56^**^			4.86^**^		3.76^*^		2.45		1.35		4.29^**^	
8–10	UW	83	40	92.1	14.90	88.1	17.96	109.3	12.56	84.8	19.12	93.8	18.56	43	82.5	13.45	90.1	14.94	90.4	17.99	77.9	13.93	87.9	18.43
	NW	569	293	91.1	15.68	86.6	16.04	105.6	16.90	85.5	17.67	95.1	20.4	276	87.0	15.54	92.3	17.37	94.0	17.52	82.0	15.54	91.8	20.09
	OW	238	129	85.9	16.78	83.5	15.57	102.3	18.19	80.8	17.16	90.4	20.73	109	80.3	14.56	82.6	15.32	89.27	16.96	79.7	15.46	87.5	21.01
	OB	121	60	77.9	14.83	75.3	14.78	99.2	17.98	77.3	15.63	80.1	18.60	61	74.1	16.41	78.9	16.54	84.7	17.50	75.6	16.24	81.2	23.40
	F (*d.f*. = 3)			13.45^***^		9.32^***^		4.10^**^		4.83^**^		9.57^***^			14.17^***^		16.39^***^		5.66^***^		3.39^*^		4.83^**^	
11–13	UW	30	19	92.9	16.51	89.5	15.11	105.5	20.63	88.3	19.47	95.6	16.65	11	73.8	16.42	85.8	20.61	85.3	10.35	64.6	16.01	84.4	25.53
	NW	318	175	87.5	14.06	95.3	17.74	109.9	17.30	69.4	22.06	87.3	22.76	143	81.3	14.67	97.4	18.38	96.3	16.92	67.8	19.21	81.1	24.12
	OW	84	57	78.0	18.58	84.0	20.38	96.9	19.88	66.9	19.74	84.5	23.29	27	73.7	16.41	91.7	22.66	87.1	20.39	63.8	17.26	76.6	26.06
	OB	15	11	71.1	18.58	72.5	17.76	97.6	11.00	63.2	18.4	78.0	25.78	4	72.8	18.03	91.5	31.10	88.5	6.25	59.0	8.37	77.5	27.8
	F (*d.f*. = 3)			10.61^***^		9.88^***^		8.33^***^		5.50^**^		1.73			2.81^*^		1.76		3.46^*^		0.67		0.37	

*MQ, motor quotient; WB, walking backward; JS, jumping sideways; MS, moving sideways; HH, hopping for height; UW, underweight; NW, normal weight; OW, overweight; OB, obesity; d.f., degrees of freedom;*

* *p < 0.05;*

** *p < 0.01;*

*** *p < 0.001. F-values were estimated through the one-way ANOVA*.

### Analyses of Motor Coordination Levels Across Age Groups by Gender and Weight Status

The one-way ANOVA performed to assess the significance level of the differences in MQ across age groups by gender and weight status (Table 4) showed that there was no difference across age groups in boys in UW, while this difference was found out in girls. Girls in OW and with OB had not significantly different MQ across age groups. In participants in NW, for both boys and girls, significantly higher performances were observed in 6–7-year-old children than 8–10 and 11–13 (*F* = 12.65, *p* < 0.001 and *F* = 8.55, *p* < 0.001, respectively). The same trend was found out in participants in OW and with OB, whose performances were better in younger groups (*F* = 8.81, *p* < 0.001 in OW category, and *F* = 5.29, *p* < 0.01 in OB category). With regard to the subtest scores, mostly the same trends with the highest significant differences were found out in the NW categories for all the subtests (except for the JS in girls), and in particular for the MS subtest (*F* = 64.97, *p* < 0.001 in boys in NW, and *F* = 57.07, *p* < 0.001 in girls in NW).

**Table 4:** F-values and significance levels of differences in the total MQ and subtest scores of the KTK across age groups by gender classes and weight status categories.

	**Across age groups**									
	**Boys**					**Girls**				
**Weight status categories**	**MQ**	**WB**	**JS**	**MS**	**HH**	**MQ**	**WB**	**JS**	**MS**	**HH**
UW	0.37	0.12	1.38	1.05	3.32^*^	3.67^*^	1.04	0.61	10.93^***^	0.18
NW	12.65^***^	15.13^***^	4.70^**^	64.97^***^	24.55^***^	8.55^***^	6.51^**^	1.19	57.07^***^	12.77^***^
OW	8.81^***^	0.04	3.39^*^	17.81^***^	5.46^**^	2.79	3.98^*^	0.35	15.77^***^	3.89^*^
OB	5.29^**^	3.01	0.81	6.25^**^	3.25^*^	0.88	1.29	0.74	5.67^**^	0.10

*MQ, motor quotient; WB, walking backward; JS, jumping sideways; MS, moving sideways; HH, hopping for height; UW, underweight; NW, normal weight; OW, overweight; OB, obesity;*

* *p < 0.05;*

** *p < 0.01;*

*** *p < 0.001. F-values were estimated through the one-way ANOVA*.

### Analyses of Motor Coordination Levels Across Gender Classes by Age and Weight Status

The one-way ANOVA carried out to assess the significance level of the differences in MQ across gender classes by age and weight status (Table 5), highlighted that 6–7-year-old boys had better performances than girls' peers in NW, OW, and OB categories (*F* = 18.10, *p* < 0.001; *F* = 14.50, *p* < 0.001; *F* = 4.63, *p* < 0.05, respectively). This trend was similar in 8–10-year-old children in NW and OW, but not in the OB category, while it was observed only for the NW category in the 11–13 years-old group (Table 5). No differences were found out across gender categories for the WB subtest, except for the age group 8–10 (*F* = 16.60, *p* < 0.001). For the JS subtest, there were strong differences in all weight status categories and age groups, with the only exception of 11–13-year-old adolescents with OB, who did not differ in their performance between boys and girls (*F* = 2.40, *p* > 0.05). The MS subtest did not reveal any significance in all classes, except for 8–10-year-old children in NW and 11–13-year-old adolescents in UW. The HH subtest showed strong differences only in the age group 6–7, and small significant difference in 11–13-year-old adolescents in NW.

**Table 5:** *F*-values and significance levels of differences in the total MQ and subtest scores of the KTK across gender classes by age groups, and weight status categories.

	**Across gender classes**														
	**6–7**					**8–10**					**11–13**				
**Weight status categories**	**MQ**	**WB**	**JS**	**MS**	**HH**	**MQ**	**WB**	**JS**	**MS**	**HH**	**MQ**	**WB**	**JS**	**MS**	**HH**
UW	3.64	2.53	7.17^*^	0.64	11.30^**^	9.53^**^	0.31	30.30^***^	3.58	2.05	9.34^**^	0.31	9.12^**^	11.69^**^	2.13
NW	18.10^***^	0.74	18.10^***^	3.26	49.71^***^	9.83^**^	16.60^***^	65.08^***^	6.14^*^	3.71	14.70^***^	1.09	49.17^***^	0.42	5.64^*^
OW	14.50^***^	1.54	24.06^***^	0.91	30.41^***^	7.59^**^	0.18	32.35^***^	0.25	1.14	1.25	2.42	4.45^*^	0.50	1.98
OB	4.63^*^	0.23	10.21^**^	0.07	6.91^*^	1.76	1.59	20.25^***^	0.36	0.08	0.02	2.28	2.40	0.19	0.00

*MQ, motor quotient; WB, walking backward; JS, jumping sideways; MS, moving sideways; HH, hopping for height; UW, underweight; NW, normal weight; OW, overweight; OB, obesity;*

* *p < 0.05;*

** *p < 0.01;*

*** *p < 0.001. F-values were estimated through the one-way ANOVA*.

### Interaction Effect Analysis of Weight Status, Age, and Gender on Motor Coordination

**Figure 1:**
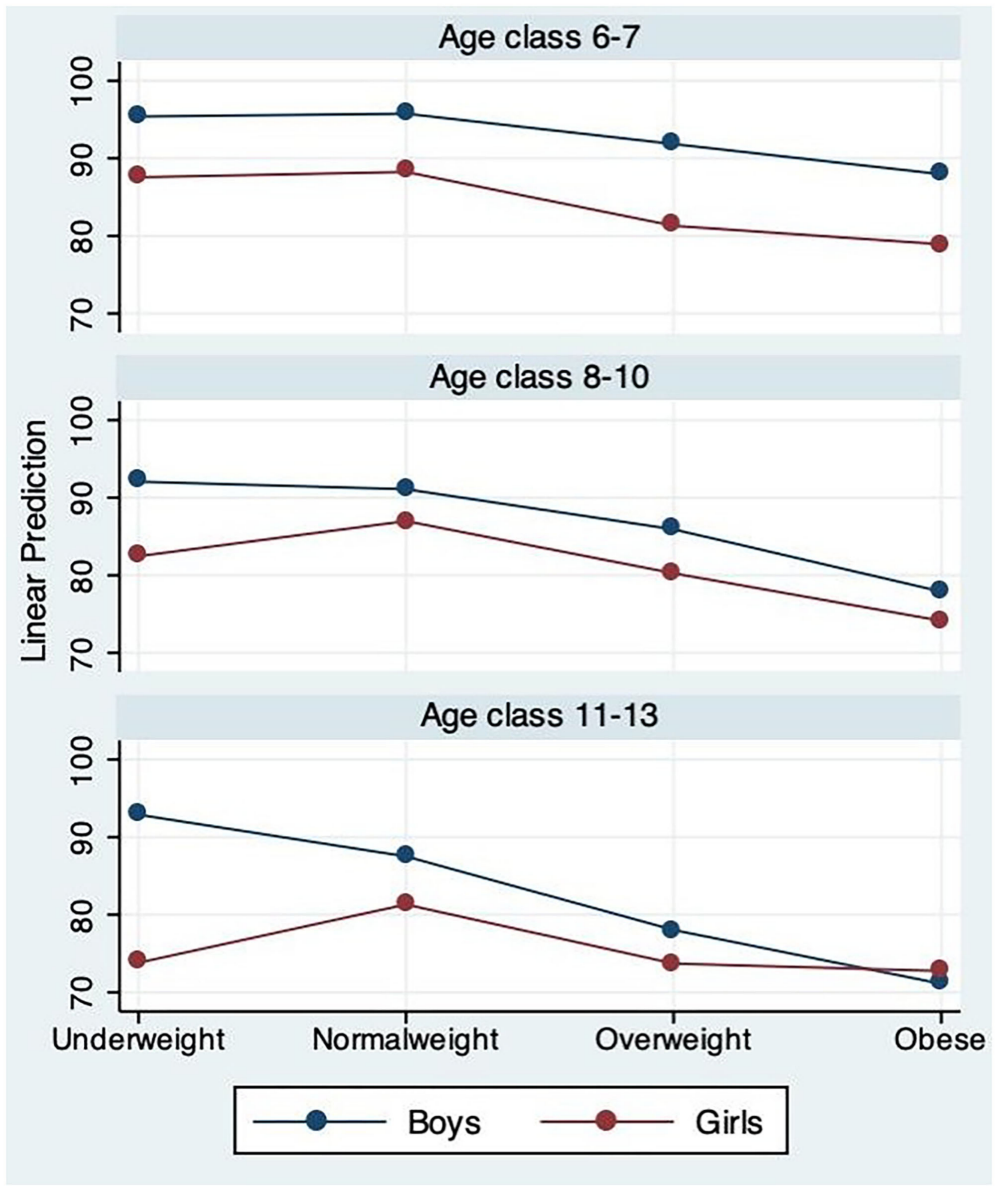
Adjusted predictions of weight status-age-gender on MQ.

The three-way ANOVA evidenced no interaction effect of weight status, age, and gender on MQ scores (Table 6 and Figure 1). Thus, it means that the joint effect of weight status, age, and gender on MQ is not statistically higher compared to the sum of the three effects individually.

**Table 6:** Interaction effect of weight status, age, and gender on MQ.

***N*** **= 1961**	***R*** **^2^ = 0.1257**			
**MQ**	**Partial SS**	**d.f**.	**F**	***p*** **-value**
Weight status	18145.61	3	25.96	0.0000
Gender	8460.35	1	36.32	0.0000
Age	8306.30	2	17.83	0.0000
Weight status × gender	1330.26	3	1.90	0.1270
Weight status × age	969.75	6	0.69	0.6547
Gender × age	429.91	2	0.92	0.3976
Weight status × gender × age	1182.53	6	0.85	0.5343

*MQ, motor quotient; SS, sum of squares; d.f., degrees of freedom. F-values were estimated through the three-way ANOVA*.

## Discussion

The aim of the present study was to investigate the association between motor coordination (MC), expressed as motor quotient (MQ), and body weight status in Italian children and early adolescents aged 6–13 years according to age and gender. Similarly, we analyzed the associations between the four motor skills included in the test protocol for the MC assessment and weight status categories across age groups and gender classes.

First of all, it should be noted that the prevalence of participants in UW, NW, OW, and with OB was 8, 61, 22, and 9%, respectively, that is a proportion which is consistent with recent data on Italian children of the same age (39). Based on this premise, our hypothesis was mostly confirmed because the findings showed that weight status, age, and gender were strongly associated with MC. In fact, MQ level decreased (*p* < 0.001) from participants in OW to participants with OB (*F* = 37.66), in older than younger (*F* = 25.25), and in girls than boys (*F* = 67.22). Lower levels of MQ, HH, JS, WB, and MS (*F* = 128.41) were found in participants in OW and with OB than peers in NW, both in boys and girls, and in all the age groups except for the age group 6–7-year-old boys.

This latter result is consistent with outcomes obtained in our previous study, in which gross motor quotient, locomotor, and object control skills were not significantly different throughout the classes of underweight, normal overweight, and obesity in preschool children aged 3–5 years old (40). Therefore, MC performances appears to not be significantly affected by weight status in early childhood as also demonstrated in the present study by the highest levels of MQ scores in 6–7-year-old children. These findings could be explained with the relevant range of interindividual variation in early motor development. Indeed, development process can be continuous but also intermittent when periods of instability or negligible changes are followed by significant progresses, or when regression stages precede a more advanced stage (41). As a matter of the fact, according to the “reciprocal interweaving” model, during childhood and adolescence the development of motor competence can alternate periods of maturity followed by subsequent periods of immaturity, and so on (42).

Our findings relating to the negative influence of higher BMI levels on MC are in agreement with previous studies in which it was detected that children and adolescents in overweight or with obesity were more likely to possess lower MC than peers in healthy weight (3, 12, 21).

The lower MQ levels in older than younger ones (*F* = 25.25) could be also associated with the high prevalence of participants in OW and with OB in 8–10 and 11–13 age groups. In agreement with previous studies (9, 21), the reported deficiencies in MC associated with OW and OB do not seem to be temporary, and we found that, increasing age, MC even appeared to deteriorate. This could be related to a disadvantageous effect of an excessive body mass on MC. In fact, it increases as a greater body mass is involved in the action and when the body needs to be moved under time constraints or against gravity (8).

Our results showed significant differences in MQ (*F* = 67.22) and JS scores (*F* = 268.38) between girls and boys. These results are similar to those reported in other studies that exhibited significant differences in motor skills between boys and girls (9, 43). Gender differences in motor performance, in which boys performing better than girls, could be related to the difference both in the usual PA level and the sport practice, regardless of body weight status (9, 44, 45). Indeed, in a cohort of 2,815 children and adolescents of both sexes aged 3–15 years, in which ~ 90% of the participants had a healthy body weight status, Ishii et al. (45) reported that boys were more physically active than girls (45). Similar results were also found by Ridley et al. (46) in the practice of organized sports in which authors, among the findings, detected that between girls' and boys' soccer teams, the latter spent significantly higher time in moderate to vigorous physical activity (MVPA) (46). In a similar way, previous studies reported lower time spent in MVPA among children with obesity than peers without obesity (47).

The significant (*p* < 0.001) inverse trend revealed for the WB subtest, with a significant increasing performance in older participants (*F* = 12.64) and in girls (*F* = 17.47), is in agreement with seminal studies on this topic. For instance, D'Hondt et al. (9) detected increasingly higher scores in this motor skill with rising age in children, as well as in girls than boys of the same age in all weight categories (9). This outcome could be related to the better balance performance of adolescents than children (48), and that of girls than same-aged boys, regardless of body weight status (49–51). According to Woollacott and Shumway-Cook (48), we speculate that older participants exhibit better dynamic balance in WB subtest than younger ones because the maturation of balance control does not complete in childhood, but, possibly, continues throughout adolescence (52). However, the literature reports contrasting results on the impact of body weight on balance control in children and adolescents (53, 54).

No interaction effect was found between weight status, age, and gender on MQ. Thus, it means that the joint effect of weight status, age, and gender on MQ is not statistically higher compared to the sum of the three effects individually. For example, a girl in OW, who is also in the higher age group, has a lower performance, but, since there is not any interaction between being girls, OW, and belonging to the higher age groups, the effect on decreasing performance is not enhanced compared to the sum of the single aspects.

In conclusion, these data showed that weight status affects the development of MC throughout childhood and early adolescence.

### Perspective

It is known that children's and adolescents' general behavior, including daily activities and the practice of PA, is determined by MC (7). Furthermore, higher levels of MC during childhood and adolescence influence children's ability to successfully participate in movement situations and, moreover, to engage in lifelong PA (15–17). Since sedentary behaviors and a low level of PA negatively affect body weight across childhood and adolescence (25, 40, 55, 56), it is important to take into account MC in children and adolescents with excessive BMI.

Therefore, the adoption of a health-related educational strategy, such as motor plans that include the development of motor coordination, is crucial in school in order to promote an active and healthy lifestyle in children and adolescents (57–60).

### Strengths and Limitations

The main strength of the study is the large Italian sample recruited. Furthermore, among the strengths it should be noted that not only the general level of MC was taken into consideration but also the score of each subtest.

Growth and maturity characteristics of children (morphological, physiological, and neuromuscular) might contribute to influence the development of MC during childhood and adolescence, therefore, the absence of data concerning these aspects represents a limitation of this study. Additionally, it is important to highlight among the limitations of the study that, although KTK is a reliable protocol test commonly used to measure MC performance in children and adolescents, it does not include the assessment of fine motor coordination.

## Data Availability Statement

The raw data supporting the conclusions of this article will be made available by the authors, without undue reservation.

## Ethics Statement

The studies involving human participants were reviewed and approved by Ethical Board of Verona University (No. 2019-UNVRCLE-0298910) and Palermo University (No. 8/2019). Written informed consent to participate in this study was provided by the participants' legal guardian/next of kin.

## Author Contributions

ML, MB, LG, and CB conceptualization and methodology. VB, MG, MCG, and AG Data collection. GT, GB, and MB data analysis. VG, ML, FS, and AP data interpretation. GB and VG writing—original draft preparation. MB writing—review & editing. MB and AP supervision. All authors have read and approved the final version of the manuscript, and agree with the order of presentation of the authors.

## Conflict of Interest

The authors declare that the research was conducted in the absence of any commercial or financial relationships that could be construed as a potential conflict of interest.

## Publisher's Note

All claims expressed in this article are solely those of the authors and do not necessarily represent those of their affiliated organizations, or those of the publisher, the editors and the reviewers. Any product that may be evaluated in this article, or claim that may be made by its manufacturer, is not guaranteed or endorsed by the publisher.
